# Topographic connectivity reveals task-dependent retinotopic processing throughout the human brain

**DOI:** 10.1073/pnas.2017032118

**Published:** 2020-12-28

**Authors:** Tomas Knapen

**Affiliations:** ^a^Spinoza Centre for Neuroimaging, Royal Netherlands Academy of Sciences, Meibergdreef 75, 1105 BK Amsterdam, The Netherlands;; ^b^Cognitive Psychology, Faculty of Behavioural and Movement Sciences, Vrije Universiteit, Van der Boechorststraat 7, 1081 BT Amsterdam, The Netherlands

**Keywords:** retinotopy, hippocampus, connective field, population receptive field, naturalistic vision

## Abstract

The human visual system is organized as a hierarchy of maps that share the topography of the retina. Known retinotopic maps have been identified using simple visual stimuli under strict fixation, conditions different from everyday vision which is active, dynamic, and complex. This means that it remains unknown how much of the brain is truly visually organized. Here I demonstrate widespread stable visual organization beyond the traditional visual system, in default-mode network and hippocampus. Detailed topographic connectivity with primary visual cortex during movie-watching, resting-state, and retinotopic-mapping experiments revealed that visual–spatial representations throughout the brain are warped by cognitive state. Specifically, traditionally visual regions alternate with default-mode network and hippocampus in preferentially representing the center of the visual field. This visual role of default-mode network and hippocampus would allow these regions to interface between abstract memories and concrete sensory impressions. Together, these results indicate that visual–spatial organization is a fundamental coding principle that structures the communication between distant brain regions.

## Significance

Vision is organized retinotopically—according to the reference frame of the retina. How much of the brain is retinotopically organized remains unknown, because traditional retinotopic mapping experiments require strict fixation and sparse stimuli. Conversely, in everyday vision we use eye movements and interaction, to derive meaning from our complex surroundings. Here, I discover retinotopic activations by explaining brain-wide BOLD signals during several experiments in terms of the pattern on the surface of primary visual cortex. This revealed visually organized processing also in regions outside the visual system, in brain regions traditionally thought devoted to memory. This visual organization in default-mode network and hippocampus speaks to the joint operation of sensations and memory in everyday vision and mental life.

Our experience of the world is ultimately based on impressions arriving through the senses. In our dominant sensory modality, vision, processing is retinotopic: organized according to the layout of the retina (1). That is, neighboring locations in the brain represent neighboring locations in the visual field. Retinotopic mapping experiments leverage sparse visual stimulation during fixation, allowing researchers to relate the elicited brain responses to visual space and delineate retinotopic maps in the brain (2, 3). Yet, in everyday life visual inputs are not sparse, and naturalistic vision is characterized by continuous eye movements and dynamic cognitive demands. It is therefore likely that charting especially high-level visual function falls outside the scope of traditional retinotopic mapping experiments.

**Fig. 1.**
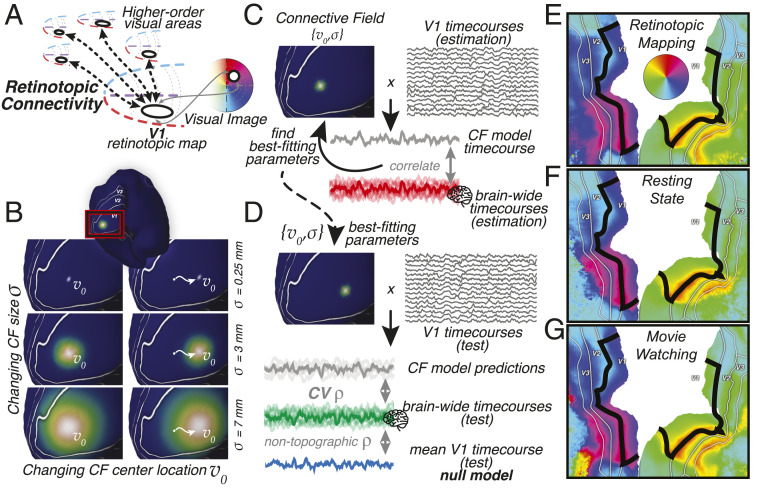
(*A*) Visual processing in higher-order brain regions reveals itself through spatially specific retinotopic connectivity with V1. V1’s map of visual space allows us to translate retinotopic connectivity to representations of visual space. (*B*) Retinotopic connectivity is quantified by modeling responses throughout the brain as emanating from Gaussian connective fields on the surface of V1. Example Gaussian CF model profiles with different size (σ) and location ($v_{0}$) parameters are shown on the inflated surface. (*C*) Predictions are generated by weighting the ongoing BOLD signals in V1 with these CF kernels. Estimating CF parameters for all locations throughout the brain captures significant variance also outside the nominal visual system (non-V1 time courses). (*D*) Cross-validation procedure. CV prediction performance of the CF model is assessed on a left-out, test dataset and is corrected for the performance of a nontopographic null model, the average V1 time course. (*E–G*) CF modeling results can be used to reconstruct the retinotopic structure of visual cortex. Polar angle preferences outside the black outline are reconstructed solely based on their topographic connectivity with V1, within the black outline. CF modeling was performed on data from retinotopic-mapping, resting-state, and movie-watching experiments separately. Visual field preferences are stable across cognitive states, as evidenced by the robust locations of polar angle reversals at the borders between V2 and V3 field maps. Additional retinotopic structure visualizations are in *SI Appendix*, Figs. S1 and S2.

Retinotopic processing throughout the brain can be identified based on topographically specific connectivity with V1 (4–6), the first visual region of the cerebral cortex (Fig 1*A*). There are several distinct advantages to assessing visual processing by means of retinotopic connectivity (RC), in which responses throughout the brain are explained in terms of the spatial pattern of activation on the surface of V1. First, RC is robust in the face of eye movements, because its reference frame is fixed in the brain and not the outside world. Second, because V1 harbors a map of visual space, RC patterns throughout the brain can be translated back into visual space coordinates. In effect, RC allows us to project the retinotopy of V1 into the rest of the brain. Finally, since brain responses are explained as a function of ongoing activations, RC can be estimated for any experimental paradigm. Thus, RC can be used to compare detailed visual–spatial processing across experiments and cognitive states.

I performed RC analysis on the Human Connectome Project (HCP) 7T dataset of 174 subjects in which data were collected during retinotopic-mapping, resting-state, and movie-watching experiments. This allowed the identification of previously unknown visual–spatial processing throughout the brain, and the quantification of how visual space is represented—even in brain regions not traditionally considered visual. Moreover, these analyses reveal how visual representations depend on cognitive state.

## Results

A parsimonious computational model for RC posits that responses arise from a localized Gaussian patch on the surface of V1 (Fig. 1*B*), its connective field (CF) (5). One fits the CF model by comparing model time-course predictions to ongoing blood oxygenation level dependent (BOLD) response time courses throughout the brain (Fig. 1*C*). To ensure that the model captures only spatially specific topographic connectivity, I correct cross-validated (CV) model prediction performance for correlation with a nontopographic null model. This null model prediction, V1’s average time course (Fig. 1*D*), corrects for responses driven by arousal, overall contrast, or feature energy. Translating the best-fitting CF parameters into visual field locations reveals the structure of visual field maps in V2, V3, and beyond (Fig. 1 *E*–*G*). Retinotopic maps resulting from retinotopic-mapping, movie-watching, and resting-state acquisitions are similar, with their borders in the same location. This means that in low-level visual cortex the structure and strength of this retinotopic connectivity are both stable across participants and robust against variations in experimental task, stimulation, and cognitive state.

**Fig. 2.**
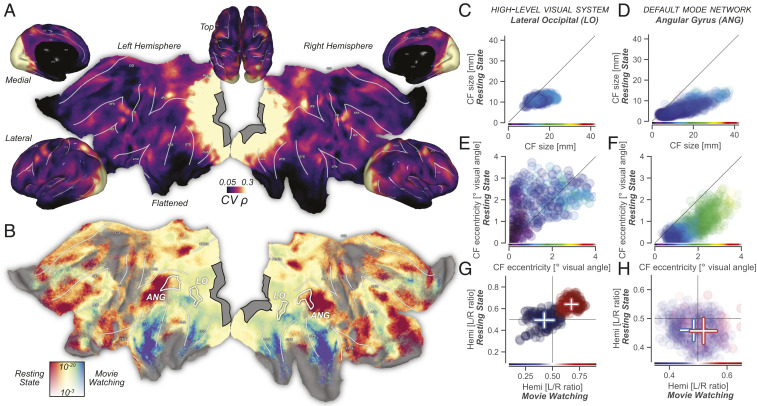
(*A*) Retinotopic connectivity during movie watching explains significant amounts of variance throughout the brain. The gray region contains all possible CF center locations; all vertex locations outside V1 that are not black have significant best-fitting cross-validated correlations. (*B*) Retinotopic connectivity is modulated by cognitive state. The local strength of topographic connectivity to V1 depends on whether participants were engaged in movie watching or endogenous thought. Ventral and lateral visual regions sensitive to object identity and responsible for audiovisual integration are more strongly connected to V1 during movie watching. Topographic connectivity between default-mode network regions and V1 is strongest during resting state. Colormap for resting state vs. movie watching represents normalized correlation ratio (*Materials and Methods*) and ranges from 0.1 to 0.9. Vertical color scale axis represents *P* values. (*C–H*) Scatter plots show CF and CF-derived visual field parameters for three experimental conditions. Horizontal and vertical axes represent movie-watching and resting-state results, respectively. Marker color represents parameter values from the retinotopy experiment and is defined on the same range as the scatter plots. Marker opacity linearly relates to null-model–corrected correlation values averaged across experimental conditions. (*C*) In the high-level visual system, CF size correlates strongly between conditions. Specifically, CF size is fixed and similar during resting-state and retinotopic-mapping experiments, with CF size variation during movie watching showing a strong correlation in CF size between conditions. (*D*) Like in LO, in the DMN the degree of spatial integration quantified by CF size is stable, with high correlations between conditions. (*E*) In LO, CF-derived eccentricity correlates strongly across conditions, and we see marked foveal biases in visual field preference for movie-watching and retinotopic-mapping experiments relative to resting state. (*F*) During resting state, CFs in the ANG DMN region become more foveal—a pattern opposite to the cognition-driven shifts of CF spatial positions in LO. During visual stimulation, the DMN represents visual space similarly to high-level visual regions (17). (*G*) In LO, visual representations are strongly contralateral and highly similar between experimental conditions (L/R: left/right). (*H*) During retinotopic-mapping and movie-watching experiments, ANG maintains a contralateral visual field preference. During resting state this tendency disappears, likely as a result of the large foveal bias in its sampling of the V1 surface.

Does this RC extend beyond the lower levels of the visual system, and, if so, how does it depend on the different cognitive states evoked in different experiments? Indeed, Fig. 2*A* shows that significant portions of movie-watching BOLD fluctuations throughout the cerebral cortex are explained as resulting from RC. That is, more than half of the cerebral cortex, including large swaths of the temporal and frontal lobes, shows significant topographically specific connectivity with V1. Interestingly, the local strength of RC depends heavily on the experiment (Fig 2*B*). During movie watching, mainly ventral visual and temporal brain regions exhibit RC. This retinotopic connectivity likely reflects object identity-related processing and audiovisual integration (7). Conversely, during resting-state scans the default-mode network (DMN) shows stronger RC, which may reflect endogenous mental imagery during mind wandering (8).

If retinotopic visual processing is a stable organizational property, visual field preferences derived from one experiment should predict RC from another. The detailed inspection of CF parameters can provide insights into the visual–spatial processing embodied by RC, but also allows us to understand its modulation by cognition. We can compare two regions, lateral–occipital (LO) and angular gyrus (ANG), as exemplars of high-level visual and DMN areas, respectively. In both regions, spatial sampling extent, as quantified by CF size, is stable (9) and precise (5, 10) during both retinotopy and resting state and becomes larger and more variable during movie watching (Fig 2 *C* and *D*). Sampling extent is strongly correlated between conditions (all linear correlations ρ>0.36, all $P<10^{-13}$ (LO), ρ>0.75, all $P<10^{-37}$ (ANG)—full statistics in *SI Appendix*, Table S1). This points to stable sampling of retinotopic space in both high-level visual and DMN regions.

In LO, the preferred eccentricity of cortical locations (Fig. 2*E*) is strongly correlated between experimental conditions (linear correlations ρ>0.48, all $P<10^{-19}$), confirming its well-known retinotopy (9, 11) (Fig. 2*C*). The detailed differences in spatial representations between conditions, however, show the flexibility of LO’s retinotopy: A very foveal bias during retinotopic mapping (11) gives way to broader coverage of the visual field during resting state and movie watching. The across-condition robustness of ANG eccentricity (Fig. 2*F*) is greater than that of LO (all linear correlations ρ>0.75, all $P<10^{-40}$). However, ANG shows an opposite pattern of retinotopic flexibility, with visual field preferences being more foveal specifically during the resting state. These foveopetal and foveofugal changes of eccentricity in both LO and ANG mimic the effects of top–down attention (12, 13) and imagination (14) on visual field representations. This pattern is also similar to what happens to visual items of interest as they are foveated through eye movements (15) and follows the topographic distribution of feedback related to visual object information (16) in the absence of eye movements.

V1 represents contralateral visual field locations, allowing us to use the hemisphere of the best-fitting CF as a proxy for visual field representation laterality. In LO, visual representations are strongly contralateral and strongly correlated between experimental conditions (*t* test between hemispheres: all T(>123)>10, $P<10^{-19}$). In the ANG region of the DMN, there is a significant contralateral bias of visual field representations during retinotopic mapping and movie watching (all T(199)>6, $P<10^{-9}$), but, presumably due to the strong foveal bias of visual field representations, not during resting state (T(199)=0.14, P=0.9). These findings confirm previous reports that the DMN represents visual space similarly to high-level visual brain regions in situations of visual stimulation (17).

Although it is not generally implicated in traditional vision science experiments, tracer-based connectivity studies place the hippocampal formation at the top of the visual-processing hierarchy (18). Hippocampus is thought to implement the interaction between memory-related and sensory processing or imagery (19–22), leading me to reason that both narrative understanding of naturalistic inputs and internally generated thought should evoke strong RC in hippocampus, over and above its recently discovered contralateral visual field preference during retinotopic mapping (23) (*SI Appendix*, Fig. S4).

**Fig. 3.**
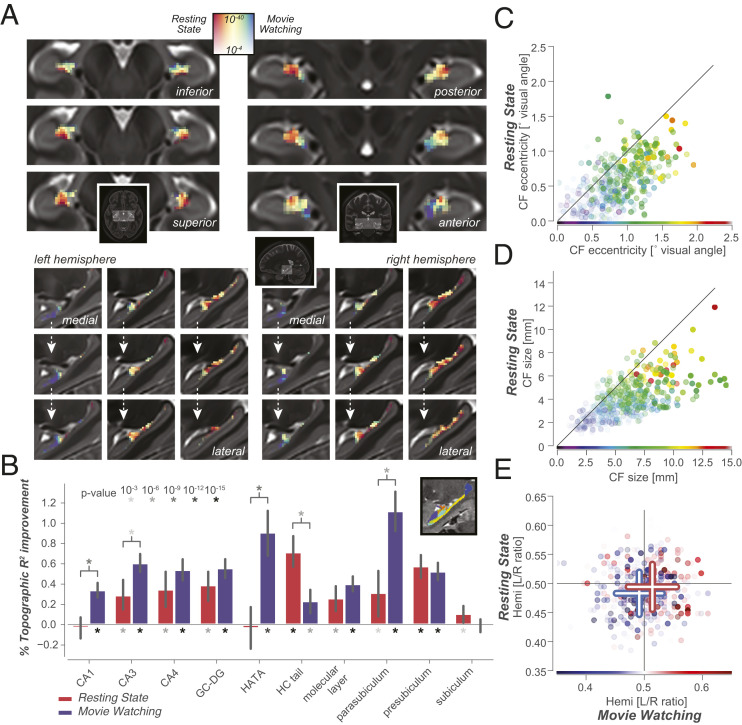
RC is pervasive throughout the hippocampal formation. (*A*) Like the cerebral cortex, the hippocampus also displays a gradient of endogenous/exogenous processing, with stronger topographic connectivity in rostral/medial/inferior regions during movie watching, and in caudal/lateral/superior regions during resting state. Colormap for resting state vs. movie watching represents normalized correlation ratio ranges from 0.35 to 0.65: a narrower range than that used for cortex in Fig. 2. Full lightbox visualization of RC connectivity gradient and visual field parameters are in *SI Appendix*, Fig. S4. We see RC in both the anterior and posterior portions of the hippocampus in both experiments, rendering it unlikely that signal-to-noise-ratio (SNR) differences between anterior and posterior hippocampus (23) impact these specific findings. (*B*) Strength and stability of RC for different cognitive states, for separate hippocampal subfields. *Inset* shows a sagittal section of a hippocampal subfield segmentation for a single subject. (GC-DG, granule cell layer of the dentate gyrus; HC, hippocampus.) Full statistics are given in *SI Appendix*, Tables S2–S4. (*C* and *D*) Eccentricity (*C*) and size (*D*) of hippocampal CFs are stable between different cognitive states, with strong correlations between all three conditions (linear CF eccentricity and size correlations >0.55, $P<10^{-26}$). (*E*) Hippocampal visual field representations are significantly biased to represent the contralateral visual hemifield in movie watching, resting state, and retinotopy (*t* test between hemispheres, $T=-4.3,P<2\cdot 10^{-5},df=173$, $T=-3.4,P<10^{-3},df=173$, and $T=-10.3,P<10^{-20},df=173$, respectively). These small yet significant contralateral biases are similar in scale to the contralaterality of visual field representations in the DMN. Detailed visualization of visual field representations in hippocampal and thalamic subregions is in *SI Appendix*, Fig. S5.

Applying CF modeling to hippocampus BOLD time courses (Fig. 3*A*) reveals significant hippocampal–V1 RC in all experimental conditions. The most striking feature in the hippocampus is a gradient of movie-watching vs. resting-state RC preference along both the medial/lateral and the long, anterior/posterior axes of the hippocampus. This RC gradient corresponds in detail to previously found microstructural and functional connectivity gradients (24–26). The high power, quality, and spatial resolution of both functional and anatomical HCP images allow the quantification of cognitive state-dependent RC per hippocampal subfield. A strong prediction based on earlier findings in both mice (22) and humans (27) is that the Cornu Ammonis (CA) region and uncus of the hippocampus should subserve its connectivity with visual cortex during visual stimulation.

The strength and cognitive-state dependence of RC varies strongly across hippocampal subfields (Fig. 3*B*). Specifically, RC driven by movie watching is strongest in parasubiculum, CA1, CA3, dentate gyrus (DG), and the hippocampal–amygdalar transition area (HATA) which principally represents the hippocampal uncus. Conversely, resting-state–driven RC is strongest in the presubiculum and the hippocampal tail. Focusing on the visual space representations of hippocampus, as quantified by CF model parameters, reveals strong correlations between experimental conditions (Fig. 3 *C*– *E*). Specifically, the detailed patterns of differences in visual field representations between experimental conditions closely resemble those that occur in the DMN (Fig. 2 and *SI Appendix*, Figs. S3 and S5).

## Discussion

CF modeling reveals retinotopic connectivity throughout the human brain, which can be quantified in terms of visual space representations. Moreover, as CF modeling can be applied to any ongoing timeseries data, it can be used to reveal changes in visual space representations across experiments.

Connective field models are driven by topographically specific BOLD responses, but do not distinguish between the many possible bottom–up and top–down sources of those responses. This is the power of the approach, but also causes interpretative challenges. Could these patterns of retinotopic connectivity be due to the complex spatiotemporal correlations that characterize the naturalistic visual inputs of, and engagement with, the movie-watching experiment? Indeed, based on just the movie-watching results, we cannot definitively assert that topographic connectivity is evidence for visual–spatial processing. But in the resting state, topographic connectivity arises from the brain’s internal organization. Finding similar RC patterns in resting state and movie watching thus hints strongly at a consistent visual–spatial structure that the brain has internalized and that is used in ongoing thought. Moreover, many of these spatiotemporal correlations in visual input and behavior that characterize movie watching are explicitly avoided by the retinotopic-mapping stimulus material and paradigm (for example, the fact that usually, human heads occur on top of bodies). I construe the similar patterns of RC between movie-watching and retinotopy experiments as evidence for a joint visual frame of reference used throughout the brain.

This also suggests that the degree of similarity in RC patterns between all three experiments can be seen as a gauge for how “visual” a brain region is. The gradient of RC similarity across experiments in both cortex and hippocampus (Figs. 2 and 3) aligns surprisingly well with a combination of functional connectivity gradients found previously in the resting state. Specifically, resting-state–movie-watching preferences behave as a combination of the primary sensory-transmodal and the multiple-demand gradients (24)—both in cortex and within the hippocampus (26). These large-scale gradients result from the push–pull between sensory and attentional brain systems on the one hand and the DMN on the other (28). Such countervailing activations and deactivations are thought to mediate a dynamic balance between outward-oriented, stimulus-driven processing on the one hand and memory-related, endogenously generated processing on the other (29, 30). Importantly, the present work identifies a consistent mode of organization across both memory-related and stimulus-directed processing: In both cognitive states, connectivity is retinotopically organized. Furthermore, these results demonstrate that the push–pull between DMN and sensory regions in terms of signal amplitude (28) also involves a trade-off in foveally biased processing between regions, possibly related to recent findings of retinotopic traveling waves in visual cortex during resting state (31, 32). On the basis of these findings, I propose that the detailed structure of concurrent retinotopically organized activations in visual system, DMN, and hippocampus gives rise to interactions between attentional and mnemonic processing (33).

The present finding, that the hippocampus shares a retinotopic mode of organization with much of the rest of the brain, is in line with canonical tracer-based network findings (18). As hippocampus also entertains world-centric coding of space (34), this solidifies the notion (19) that hippocampus is a nexus for the conjunctive coding of both world-centric and sensory reference frames (20, 35).

The stability of RC structure across experiments points to the brain’s use of sensory topography as a fundamental organizing principle to facilitate neural communication between distant brain regions. By casting ongoing brain responses into a common reference frame, topographic connectivity can facilitate our understanding of neural processing as resulting from canonical computational mechanisms such as divisive normalization (36). Future work will be able to leverage topographic connectivity to investigate the computational hierarchy that generates increasingly world-centric representations of space (37) and culminates in the medial temporal lobe (34, 38).

## Materials and Methods

### Human Connectome Project Data.

HCP 7T functional MRI (fMRI) data were used, in conjunction with 3T anatomical MRI images (39). In total, 2.5 h from 174 subjects with full data of all 7T experiments were used, sampled at 1.6 mm isotropic resolution and a rate of 1 Hz (40). For all functional analyses, the Fix independent component analysis-denoised time-course data, sampled to the 59,000 vertex-per-hemisphere areal feature-based cross-subject alignment method (MSMAll) surface and 1.6-mm Montreal Neurological Institute (MNI) volume formats, were used. These data are freely available from the HCP project website.

### Analysis.

Hippocampal subfield segmentation was performed using FreeSurfer, after which the individual subfields were warped to the functional data’s MNI space using the existing HCP warp fields with nearest-neighbor interpolation. High-resolution subfield segmentations were smoothed with a Gaussian of 0.8 mm σ to ensure representation of all subfields when resampled to the 1.6-mm resolution of the functional images. Anatomical region of interest (ROI) definitions were taken from the multimodal parcellation atlas (41) for surface data and FMRIB Software Library’s Jülich histological atlas (42) for hippocampal ROIs in MNI volumetric space.

Functional data of all three experiments [movie (approximately 1 h), retinotopy (43) (30 min), and resting state (1 h)] were preprocessed identically, by means of high-pass filtering (third-order Savizky–Golay filter, 210-s period) and z scoring over time.

To create a template of retinotopic spatial selectivity, I averaged the time courses of the retinotopic-mapping experiment (bar and wedge conditions) across participants and estimated linear Gaussian population receptive field (pRF) model parameters from

$$g\left(x_{0},y_{0},\sigma \right)=exp\left(-\frac{{\left(x-x_{0}\right)}^{2}+{\left(y-y_{0}\right)}^{2}}{2\sigma^{2}}\right),$$ [1]

where $x_{0}$, $y_{0}$, and σ are the parameters that define the location (in the Cartesian {x,y} plane) and size of the pRF, respectively. The fitting procedure consisted of an initial grid fit stage, followed by an iterative fitting stage using the L-BFGS-B algorithm as implemented in scipy.optimize. The identical fitting procedure was performed on the hippocampus to create figures of visual–spatial representations shown in *SI Appendix*, Fig. S4.

Gaussian connective field profiles on the surface are defined for each vertex v on the cortical manifold as

$$CF\left(v_{0},v,\sigma \right)=exp\left(-\frac{|v-v_{0}{|}^{2}}{2\sigma^{2}}\right),$$ [2]

where $v_{0}$ is the center location of the connective field, and σ is the Gaussian spread in millimeters on the cortical surface. Diffusion of heat along the fiducial, or midgray, surface mesh was simulated and then used to infer geodesic distances $\left|v-v_{0}\right|$ between all V1 vertices, as implemented in pycortex (44) (which was also used for all surface-based visualization). As the two hemispheres are two separate surface meshes, this distance matrix was calculated for each hemisphere separately. Only V1 vertices with a pRF-fit within-set $R^{2}$ of >0.2, a peak pRF position inside the stimulus aperture used in the retinotopy experiment, and a positive pRF amplitude in the above pRF analysis served as the center of a candidate CF. These conservative selection criteria were chosen to ensure that CFs are centered on visually responsive vertices within V1 and improves the interpretability of the relation between CF parameters and visual field coordinates. Separate analyses (results not reported here) served to verify that using the full V1 map as a possible CF center yielded similar RC results on the whole. Grid-fit candidate CF sizes ranged from very small (biased to the center vertex only) to evenly spanning almost the entirety of V1: [0.5, 1, 2, 3, 4, 5, 7, 10, 15, 20, 30, 40, 80] mm σ for the Gaussian CF.

The predicted time course for each of the resulting 14,287 CF models was generated by taking the dot product between the CF’s vertex profile and the vertex by time-courses matrix in V1. In this operation I did not apply the conservative selection criteria used for CF center vertices; all vertex time courses in V1 were used regardless of eccentricity, pRF-fit $R^{2}$, etc. Additionally, I ensured that CF models were based on only V1 time courses. That is, if the CF extended into V2, these time courses were not used to generate the CF model time course. The resulting CF model time courses were z scored and correlated with the time courses throughout the brain, without convolution with a hemodynamic response function. For each location in the brain, the connective field resulting in the highest squared correlation was selected. Subsequently, this specific connective field was used to generate model time courses for out-of-set predictions of left-out data. A fourfold scheme was used for cross-validation. As both the resting state and movie watching consist of four separate runs of approximately 15 min each, this scheme was implemented to be identical to a leave-one-run-out cross-validation pattern. CF parameters and correlation measures were then averaged across runs for further analysis.

The resulting out-of-set correlation was referenced against the correlation with a nonspatial null model, in which the time courses throughout the brain were predicted by the average V1 time course. This correction means that although corrected correlation values are no longer interpretable as correlations, they can serve to conservatively assess the presence of true topographic connectivity. Moreover, comparisons between conditions based on these corrected correlation values explicitly discount changes in nontopographic correlations between brain regions. One-sample *t* tests were used to compare corrected out-of-set prediction performance against 0, and reported *P* values are two sided. Correlations across vertices between CF parameters, as well as *t* tests of the differences in CF parameters across experiments (*SI Appendix*, Table S1), were calculated weighted by the null-model–corrected correlation values using the statsmodels.stats.weightstats package, which adjusts the degrees of freedom based on the applied weighting. To quantify a location’s RC preference during resting state vs. movie watching, a normalized ratio of the null-model–corrected CV correlation values was used for the respective experimental conditions $\frac{\rho_{RS}}{\rho_{RS}+\rho_{MW}}$, where RS and MW stand for resting state and movie watching, respectively. This measure takes a value between 0 and 1, where 0.5 signifies equal RC in both experimental conditions. Because the constituent parts of this ratio are corrected for null-model performance, this measure is not sensitive to possible nontopographic differences in V1 connectivity between conditions, such as arousal.

Pilot analyses revealed that the presented results are robust to drastic changes in the employed modeling procedure: Penalized regression using V1 time courses as the design matrix (45) produces highly comparable results. That is, CF fitting is also possible when releasing the Gaussian constraint that is based on the known topographic organization in the source region. CF modeling and RC estimation in general exemplify a broader category of analysis techniques in which decomposition of signals based on their local structure represents a very efficient manner of mapping all-to-all correlations between brain activations into a subspace relevant to local neural processing (46). Explicit CF modeling following the methods from Haak et al. (5) was chosen here because it performs direct estimation of meaningful CF parameters.

## Supplementary Material

Supplementary File

## Data Availability

All in-house analyses were implemented in python, using scientific python packages. The full list of dependencies for running the analyses, and the code itself, are available on GitHub, https://github.com/tknapen/HCP_Connective_Fields. The notebooks in this repository allow users to recreate all data visualizations presented here. Subcortical atlases for hippocampus (47) and thalamus (48) are available through their respective publications. fMRI/MRI data have been deposited in the Human Connectome Project (https://wiki.humanconnectome.org).
