# Supplementary material for: Topographic connectivity reveals task-dependent retinotopic processing throughout the human brain
Source: Proc Natl Acad Sci U S A. 2020 Dec 28;118(2):e2017032118. doi: 10.1073/pnas.2017032118 (PMC7812773; doi:10.1073/pnas.2017032118)
Supplement: Supplementary File [file pnas.2017032118.sapp.pdf]

PNAS
www.pnas.org

Supplementary Information for

Topographic connectivity reveals task-dependent retinotopic processing throughout the human brain

Tomas Knapen

Tomas Knapen
E-mail: tknapen@gmail.com

This PDF file includes:

Figs. S1 to S5 (not allowed for Brief Reports)
Tables S1 to S4 (not allowed for Brief Reports)
SI References

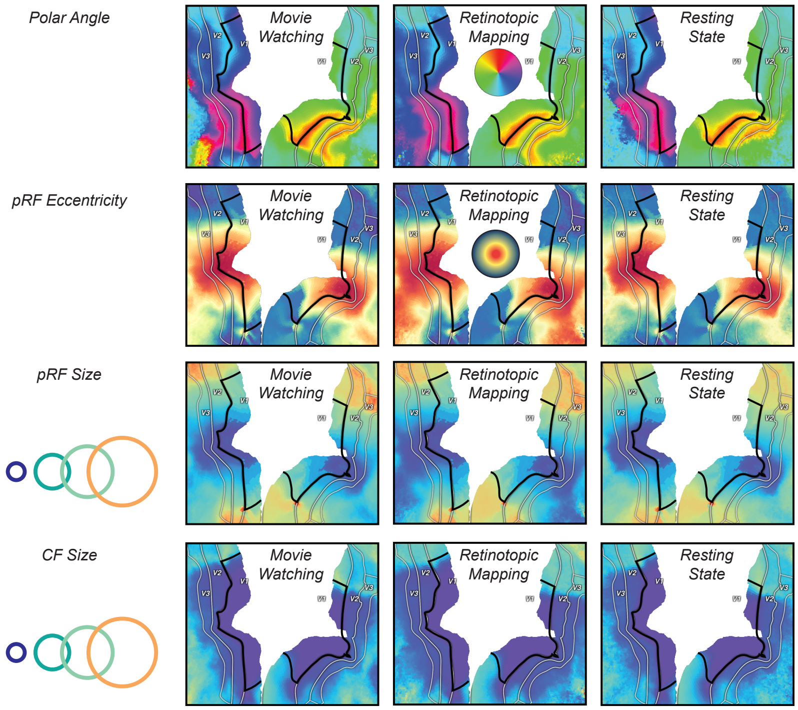

Fig. S1. Detailed visualization of retinotopic parameters resulting from CF fit in the visual system. View identical to that of Fig 1 e,f&g.

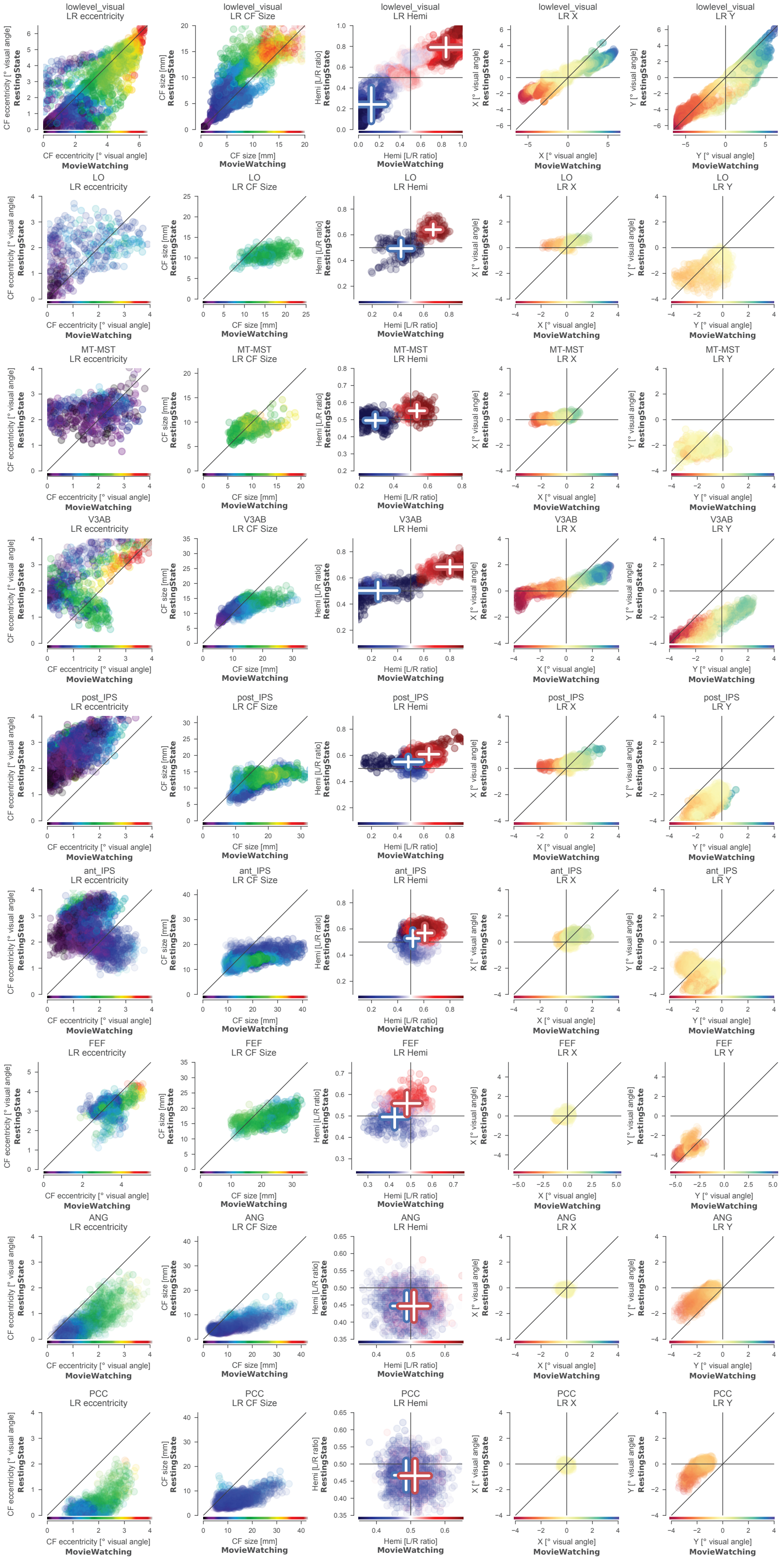

Fig. S2. CF parameter correlations between conditions for several visual regions in the dorsal visual processing stream, from lower to higher levels of processing. All ROI definitions are taken from the MMP-HCP atlas (1). "lowlevel_visual" combines V2 and V3. This figure adds columns for x and y CF-pRF parameters.

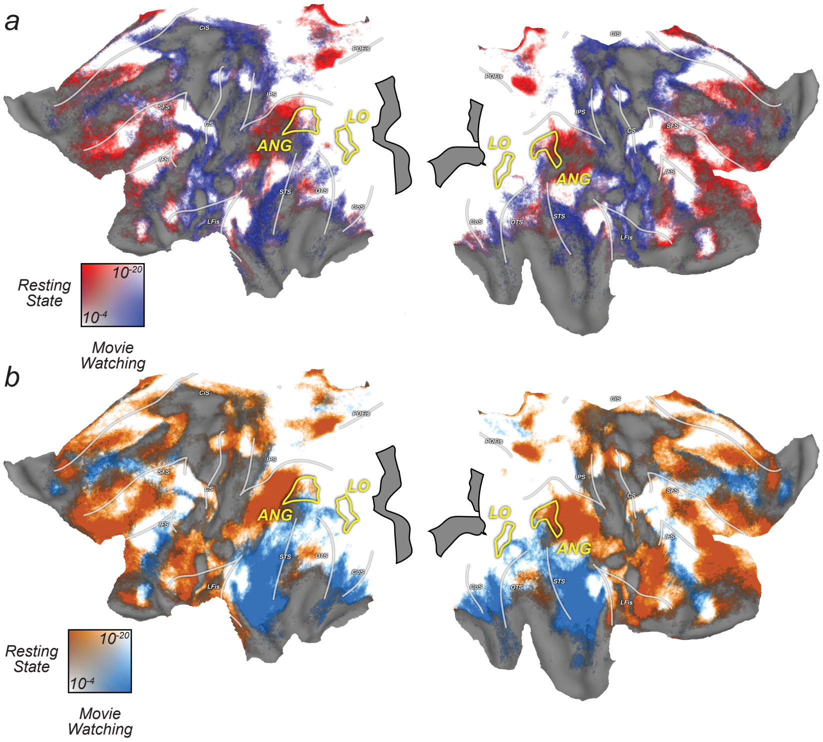

Fig. S3. CF fits are stable across experiments. **a.** It is possible to fit the CF model on one experiment, and then predict the BOLD timecourses of a second experiment. Colours represent negative $\log_{10}$ p-values for training on Resting State and testing on Movie Watching (Blue) and vice-versa (Red). In most regions (White) prediction across experiments is highly significant in both directions. Reference against the same visualization of within-experiment prediction (**b.**) reveals that the preference of a given location depends on the experiment on which it was tested. This indicates that 1. CF estimates are stable across conditions, and 2. differences in RC between experiments are driven by the strength at which RC is driven in the test, and not the train condition. CF visual field preferences are not distributed evenly across the visual field; in the horizontal direction, they tend to prefer the center of the visual field whereas in the vertical direction more CFs cover the lower meridian of visual space.

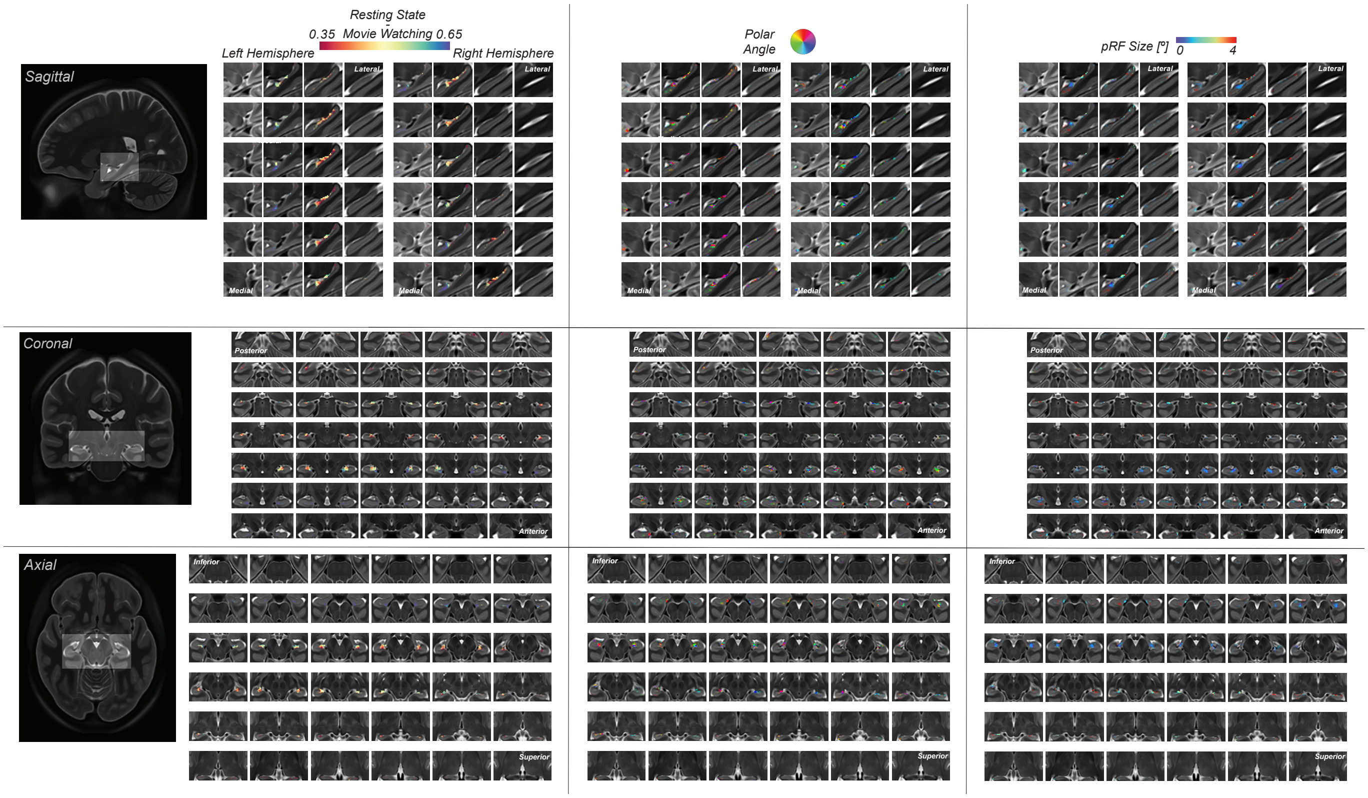

Fig. S4. Full zoomed lightbox views of relative strength of hippocampal retinotopic connectivity in Resting State and Movie Watching (left), pRF polar angle (middle) and pRF size (right), were derived from the retinotopic mapping experiment, analyzed identically to V1 (See Fig 1 & methods).

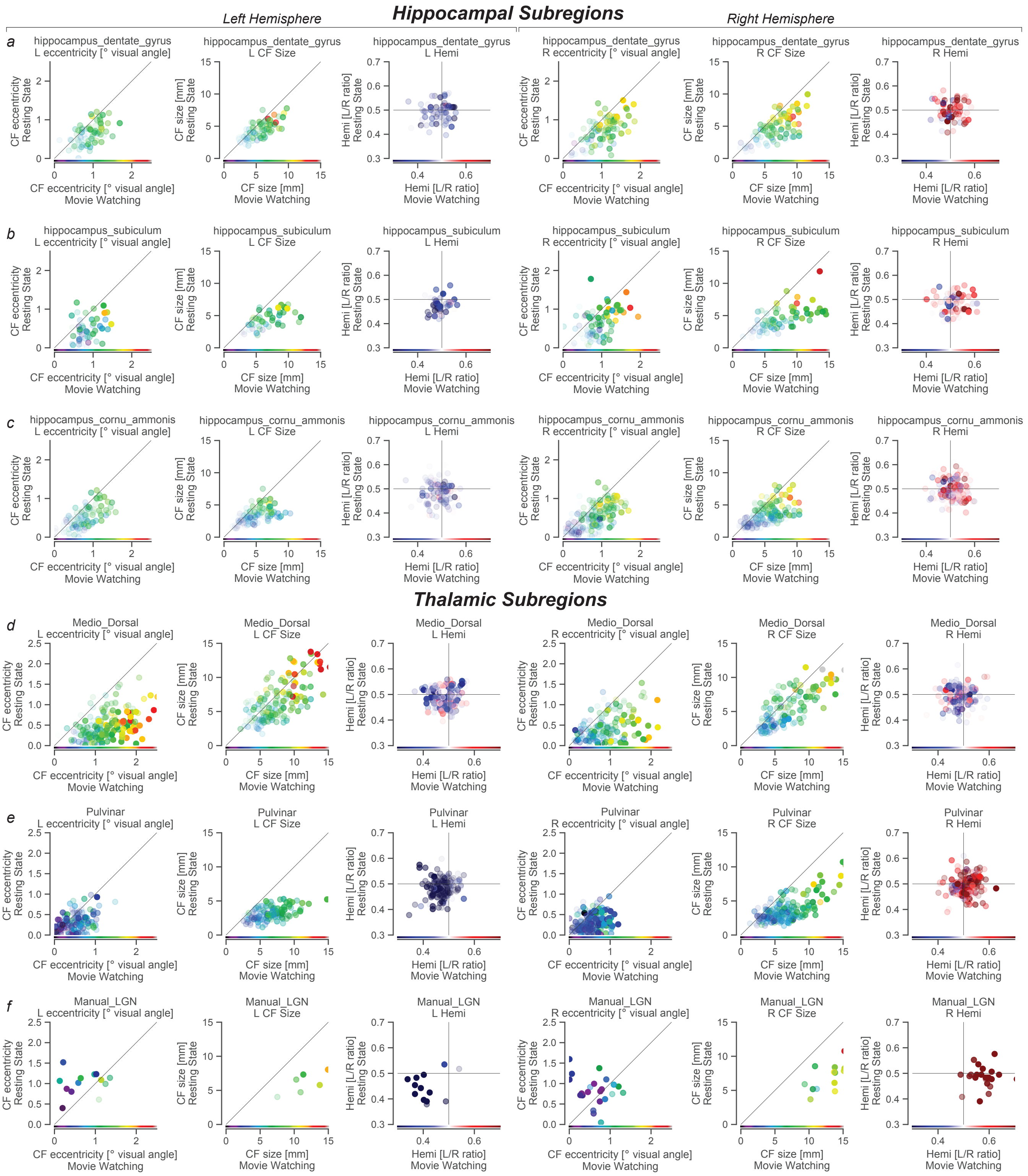

Fig. S5. CF parameters in selected subfields of subcortical (Hippocampus and Thalamus) structures, per hemisphere. ROIs taken from Jülich histological (2) and Najdenovska (3) atlases of hippocampus and thalamus, respectively, with LGN delineated manually.

Table S1. Statistics for CF parameter correlations in cerebral cortex. MW; Movie Watching, RS; Resting State, RM; Retinotopic Mapping. rho; linear correlation coefficient, wcorr; linear correlation coefficient weighted by r-squared.

FEF	V2	V3	MT-MST	V3AB	LO	post-IPS	ant-IPS	ANG	PCC
ecc MW vs RS rho	0.4498	0.9156	0.2554	0.8029	0.4470	0.7278	-0.1970	0.7923	0.6557
ecc MW vs RM rho	0.6607	0.9581	0.1751	0.7557	0.6926	0.4524	0.2978	0.6354	0.5317
ecc RS vs RM rho	0.4201	0.9089	0.3350	0.5206	0.6130	0.4794	0.2829	0.4754	0.5374
ecc MW vs RS p	0.0000	0.0000	0.0000	0.0000	0.0000	0.0000	0.0000	0.0000	0.0000
ecc MW vs RM p	0.0000	0.0000	0.0001	0.0000	0.0000	0.0000	0.0000	0.0000	0.0000
ecc RS vs RM p	0.0000	0.0000	0.0000	0.0000	0.0000	0.0000	0.0000	0.0000	0.0000
ecc wcorr MW vs RS	0.4600	0.9162	0.2170	0.7773	0.4820	0.7351	-0.1543	0.7989	0.6385
ecc wcorr RS vs RM	0.3934	0.9169	0.3304	0.4953	0.6332	0.4287	0.2667	0.5796	0.5652
ecc wcorr MW vs RM	0.6515	0.9586	0.1910	0.7420	0.7006	0.3955	0.3720	0.7078	0.5352
CF size MW vs RS rho	0.6325	0.8889	0.5090	0.6101	0.4847	0.6393	0.6523	0.8240	0.6180
CF size MW vs RM rho	-0.3419	0.9063	0.3849	0.7053	0.5863	0.3109	-0.3228	0.6763	0.2201
CF size RS vs RM rho	-0.1833	0.8884	0.4098	0.6481	0.3669	0.5121	-0.3641	0.7873	0.4611
CF size MW vs RS p	0.0000	0.0000	0.0000	0.0000	0.0000	0.0000	0.0000	0.0000	0.0000
CF size MW vs RM p	0.0000	0.0000	0.0000	0.0000	0.0000	0.0000	0.0000	0.0000	0.0000
CF size RS vs RM p	0.0000	0.0000	0.0000	0.0000	0.0000	0.0000	0.0000	0.0000	0.0000
CF size wcorr MW vs RS	0.6434	0.8808	0.5219	0.6584	0.4910	0.6467	0.6118	0.7905	0.5681
CF size wcorr RS vs RM	-0.1615	0.8858	0.4198	0.6673	0.3657	0.5113	-0.3652	0.8045	0.6365
CF size wcorr MW vs RM	-0.3085	0.9059	0.4147	0.7593	0.5898	0.3067	-0.2810	0.6091	0.3873
hemi MW vs RS rho	0.2557	0.9344	0.5372	0.8620	0.7286	0.4339	0.1413	0.1646	0.0018
hemi MW vs RM rho	0.3319	0.9504	0.9421	0.8878	0.8676	0.6810	0.5593	-0.0701	0.1709
hemi RS vs RM rho	0.6062	0.9304	0.6051	0.8423	0.7807	0.5683	0.3709	-0.1252	0.1179
hemi MW vs RS p	0.0000	0.0000	0.0000	0.0000	0.0000	0.0000	0.0000	0.0005	0.9694
hemi MW vs RM p	0.0000	0.0000	0.0000	0.0000	0.0000	0.0000	0.0000	0.1391	0.0003
hemi RS vs RM p	0.0000	0.0000	0.0000	0.0000	0.0000	0.0000	0.0000	0.0081	0.0135
hemi wcorr MW vs RS	0.2629	0.9506	0.5399	0.8723	0.7420	0.4976	0.2074	-0.0430	-0.1401
hemi wcorr RS vs RM	0.6158	0.9482	0.6115	0.8466	0.7849	0.5945	0.3816	0.0090	0.0042
hemi wcorr MW vs RM	0.3501	0.9671	0.9433	0.9027	0.8696	0.7046	0.5743	0.1360	0.1228
pRF X MW vs RS rho	0.2879	0.9225	0.5654	0.9038	0.5205	0.4566	0.2194	0.0351	-0.0206
pRF X MW vs RM rho	0.4168	0.9335	0.9320	0.9561	0.8261	0.8110	0.6049	0.0632	0.1132
pRF X RS vs RM rho	0.5405	0.8948	0.6085	0.9215	0.6590	0.5227	0.3368	-0.1130	0.2073
pRF X MW vs RS p	0.0000	0.0000	0.0000	0.0000	0.0000	0.0000	0.0000	0.4592	0.6667
pRF X MW vs RM p	0.0000	0.0000	0.0000	0.0000	0.0000	0.0000	0.0000	0.1825	0.0177
pRF X RS vs RM p	0.0000	0.0000	0.0000	0.0000	0.0000	0.0000	0.0000	0.0170	0.0000
pRF X wcorr MW vs RS	0.2998	0.9368	0.5722	0.9123	0.5226	0.5185	0.2782	-0.1261	-0.1425
pRF X wcorr RS vs RM	0.5593	0.9224	0.6124	0.9240	0.6598	0.5605	0.3437	0.0275	0.0892
pRF X wcorr MW vs RM	0.4408	0.9530	0.9356	0.9636	0.8275	0.8379	0.6203	0.0973	0.0998
pRF Y MW vs RS rho	0.4498	0.9554	0.2521	0.9503	0.4486	0.7359	-0.1967	0.8075	0.7635
pRF Y MW vs RM rho	0.6607	0.9764	0.2293	0.8950	0.7347	0.5250	0.3018	0.6354	0.5317
pRF Y RS vs RM rho	0.4201	0.9386	0.0878	0.7824	0.6138	0.4251	0.2528	0.5059	0.6346
pRF Y MW vs RS p	0.0000	0.0000	0.0000	0.0000	0.0000	0.0000	0.0000	0.0000	0.0000
pRF Y MW vs RM p	0.0000	0.0000	0.0000	0.0000	0.0000	0.0000	0.0000	0.0000	0.0000
pRF Y RS vs RM p	0.0000	0.0000	0.0540	0.0000	0.0000	0.0000	0.0000	0.0000	0.0000
pRF Y wcorr MW vs RS	0.4600	0.9568	0.2130	0.9450	0.4779	0.7411	-0.1541	0.8005	0.6925
pRF Y wcorr RS vs RM	0.3934	0.9469	0.0591	0.7642	0.6337	0.3729	0.2398	0.5748	0.6270
pRF Y wcorr MW vs RM	0.6515	0.9778	0.2835	0.8901	0.7377	0.5310	0.3741	0.7078	0.5352

Table S2. Statistics for movie-watching RC in different hippocampal subfields

Movie watching	T	dof	p-val	cohen-d	BF10	power
CA1	9.941	173	5.28584e-19	0.754	9.507e+15	1
CA3	13.855	173	3.77237e-30	1.05	8.602e+26	1
CA4	9.897	173	6.96224e-19	0.75	7.259e+15	1
GC-DG	11.367	173	5.0978e-23	0.862	8.263e+19	1
HATA	7.753	173	3.67912e-13	0.588	1.881e+10	1
HP_tail	3.813	173	9.55131e-05	0.289	163.061	0.984
molecular_layer_HP	10.848	173	1.51676e-21	0.822	2.954e+18	1
parasubiculum	10.813	173	1.90166e-21	0.82	2.366e+18	1
presubiculum	11.985	173	8.74929e-25	0.909	4.489e+21	1
subiculum	-0.35	173	0.636802	0.027	0.18	0.023

Table S3. Statistics for resting-state RC in different hippocampal subfields

Movie watching	T	dof	p-val	cohen-d	BF10	power
CA1	-0.574	173	0.716772	0.044	0.199	0.013
CA3	4.016	173	4.40624e-05	0.304	335.108	0.991
CA4	3.888	173	7.20393e-05	0.295	211.937	0.987
GC-DG	5.275	173	1.96592e-07	0.4	55870	1
HATA	-0.344	173	0.63439	0.026	0.179	0.023
HP_tail	9.485	173	9.55299e-18	0.719	5.593e+14	1
molecular_layer_HP	4.167	173	2.43291e-05	0.316	583.993	0.994
parasubiculum	2.695	173	0.00387086	0.204	5.605	0.851
presubiculum	9.74	173	1.89864e-18	0.738	2.718e+15	1
subiculum	2.585	173	0.0052737	0.196	4.267	0.824

Table S4. Statistics for the difference between resting-state and movie watching RC in different hippocampal subfields

Movie watching	T	dof	p-val	cohen-d	BF10	power
CA1	-5.87	346	1.02077e-08	0.629	970000	1
CA3	-3.748	346	0.000208772	0.402	91.057	0.962
CA4	-1.84	346	0.0666119	0.197	0.599	0.45
GC-DG	-1.889	346	0.0597003	0.203	0.654	0.47
HATA	-5.99	346	5.26794e-09	0.642	1.818e+06	1
HP_tail	4.955	346	1.13427e-06	0.531	11380	0.999
molecular_layer_HP	-1.933	346	0.0541079	0.207	0.707	0.487
parasubiculum	-5.153	346	4.31793e-07	0.552	28180	0.999
presubiculum	0.695	346	0.487632	0.074	0.149	0.107
subiculum	2.262	346	0.024307	0.243	1.366	0.616

References

1. MF Glasser, et al., A multi-modal parcellation of human cerebral cortex. *Nature* **536**, 171–178 (2016).
2. SB Eickhoff, S Heim, K Zilles, K Amunts, Testing anatomically specified hypotheses in functional imaging using cytoarchitectonic maps. *NeuroImage* **32**, 570–582 (2006).
3. E Najdenovska, et al., In-vivo probabilistic atlas of human thalamic nuclei based on diffusion- weighted magnetic resonance imaging. *Sci. Data* **5**, 180270 (2018).
